# Analysis of Endoplasmic Reticulum Stress Proteins in Spermatogenic Cells After Paclitaxel Administration

**DOI:** 10.3390/cimb47080620

**Published:** 2025-08-05

**Authors:** Suna Karadeniz Saygılı, Meryem Cansu Sahin, Fulya Yukcu, Senem Sanli

**Affiliations:** 1 Department of Histology and Embriyology, Faculty of Medicine, Kutahya Health Sciences University, Kutahya 43100, Türkiye; 2 Department of Medical Services and Techniques, Vocational School of Health Services, Usak University, Usak 64100, Türkiye; meryem.sahin@usak.edu.tr (M.C.S.); senem.sanli@usak.edu.tr (S.S.); 3 Department of Biophysics, Faculty of Medicine, Kutahya Health Sciences University, Kutahya 43100, Türkiye; fulya.yukcu@ksbu.edu.tr

**Keywords:** spermatogenesis, GC1, GC2, cell culture, endoplasmic reticulum, paclitaxel

## Abstract

Background/Objectives: The aim of this research is to analyze the effect of paclitaxel on endoplasmic reticulum (ER) stress in spermatogenic cells. Methods: In the study, spermatogonium (GC1) and spermatocyte (GC2) cell lines were used. The IC50 dose of paclitaxel was calculated using an MTT assay. Each cell line was separated into two different groups: control (GC1-C, GC2-C) and paclitaxel-treated (GC1-P, GC2-P). The control cells were incubated under standard culture conditions. The paclitaxel group cells were incubated in culture medium containing the paclitaxel IC50 dose for 24 h. After the experiments, all groups were stained with GRP78, p-PERK, and p-eIF2α antibodies using semi-quantitative immunocytochemistry. Results: Paclitaxel showed cytotoxicity. In the experimental model of the paclitaxel-treated cells, all the markers showed elevated levels of immunoreactivity, indicating ER stress. Conclusions: Paclitaxel administration triggered ER stress in spermatogenic cells. Studies of ER-related stress mechanisms in spermatogenic cells with further advanced molecular analyses will be important for therapeutic strategies.

## 1. Introduction

Paclitaxel is a commonly employed cancer treatment to combat solid tumors in breast cancer, as well as ovarian cancer and lung cancer [1]. The therapeutic action of paclitaxel depends on its ability to support microtubule growth and limit its breakdown, disrupting the normal spindle activity and resulting in cell cycle arrest and cell death. Since paclitaxel demonstrates extensive cytotoxic action on cancer cells, it simultaneously inflicts severe damage to healthy tissues, especially spermatogenic cells inside the testes, due to their high rate of cell division [2]. Research has shown that, among chemotherapeutic drugs, paclitaxel causes reproductive damage, which causes male patients to have a lower sperm count and slower sperm movement, ultimately causing infertility [3]. The pathways responsible for these negative side effects are not well-understood [4]. Chemotherapy-induced toxicity primarily affects the endoplasmic reticulum (ER) organelles, which function as the critical center for protein synthesis and folding, together with quality control operations [5]. All proteins must pass through the ER, where their correct folding and modification procedures must take place before they can reach their cellular destination. When cells are exposed to calcium disturbances, drug toxicity, and oxidative stress, misfolded or unfolded proteins are found in the ER lumen [6]. After this condition develops, ER cells begin a conserved cellular response, called the unfolded protein response (UPR), to restore ER homeostasis, inhibiting translational activities and increasing molecular chaperone production, which promotes misfolded protein destruction [7]. In summary, the UPR operates as a protective state when ER stress is extensive or severe, such that the UPR transforms from this state into a proapoptotic state [8].

The regulation of the ER stress response relies mainly on three components: Glucose-regulated Protein 78 (GRP78), PKR-like ER kinase (PERK), and phosphorylated eukaryotic initiation factor 2 alpha (eIF2α) [9]. BiP functions as GRP78 to act as a chaperone protein, which detects ER stress and triggers the UPR. Normally, PERK, IRE1, and ATF6 UPR sensors wrap around GP78 to turn off [10]. When ER stress occurs, GRP78 stops binding to these sensors, and they are thus functional and activate. Typically, this triggers PERK activation, which results in eIF2α phosphorylation and a decrease in general protein synthesis in order to reduce the ER protein burden [11]. This pathway promotes recovery by selecting stress-related protein translation and activates apoptotic signaling pathways in case permanent damage occurs. These molecular markers are essential to evaluate both ER stress initiation events and the overall stress intensity. The testis serves as the most active metabolic tissue within the body by regulating continuous cell development during spermatogenesis, through spermatogenic cell differentiation and proliferation [12]. The constantly developing state of spermatogenic cells makes these cells highly susceptible to the harmful effects of chemotherapeutic agents [13]. In vitro investigations of spermatogenesis at different developmental milestones use the established cellular models GC1 (spermatogonium) and GC2 (spermatocyte) [14]. A limited number of studies have studied the general toxicological actions of chemotherapy on male reproductive health; however, there is a lack of research on the molecular stress responses, including ER stress, within spermatogenic cells after paclitaxel treatment [15,16]. The research bridges a crucial knowledge gap regarding how paclitaxel affects ER stress signaling in germ cells, since this information matters for reproductive health and chemotherapy defense development. The rise in the number of young male patients with cancer, along with their need for protected fertility and diminished therapy-induced later effects, has made these issues critical in modern clinical oncology and reproductive medicine. This investigation focuses on how paclitaxel affects GRP78 and PERK together with phosphorylated eIF2α expression in GC1 and GC2 spermatogenic cell lines. The research team performed immunocytochemical analysis to examine the molecular stress mechanisms triggered by paclitaxel treatment in order to establish knowledge that can help develop fertility-sparing therapeutic approaches.

## 2. Materials and Methods

### 2.1. Cell Lines and Culture Conditions

Two mouse-derived spermatogenic cell lines were used in terms of spermatogonia (the early mitotic phase of spermatogenesis) and spermatocytes (the meiotic phase), GC1-spg and GC2-spd, respectively. These provide a robust in vitro testicular cell model in which to investigate testicular cell responses under experimental conditions, especially in the context of cytotoxic drug exposure. In the fully characterized medium, Dulbecco’s modified Eagle medium (DMEM), supplemented with 10% fetal bovine serum (FBS) to support optimal growth and 1% penicillin streptomycin to prevent microbial contamination, was used to culture both cell lines [17]. In a physiological environment, in terms of the cell architecture, the cells were maintained in a humidified incubator at 37 °C with 5% CO_2_. The cells were sub-cultured before reaching full confluency to ensure exponential growth and cell viability for the planned treatments. The media were changed every two days.

### 2.2. Paclitaxel IC50 Determination Using an MTT Assay

Cellular metabolic activity and viability were determined using an MTT colorimetric assay, according to the supplier’s instructions, and the half maximal inhibitory concentration (IC50) of paclitaxel for each cell line was determined. In order to ensure exponential growth and that uniform monolayers were formed, GC1 and GC2 cells were seeded into 96-well plates at an optimal density [18]. The cells were incubated for 24 h, at which point, the cells were treated with a range of paclitaxel concentrations in order to determine the dose–response curve. After an additional 24 h of incubation, the drug was allowed to interact with the treated cells. This was then followed by the addition of 10 µL of MTT reagent (at 5 mg/mL) to each well, which was then incubated for 4h. Because metabolically active cells reduce the MTT, insoluble formazan crystals were formed, which were then solubilized in dimethyl sulfoxide (DMSO). The absorption was measured at a wavelength of 570 nm using a microplate spectrophotometer. Non-linear regression analysis was used to calculate the IC50 values, determined as the concentration of paclitaxel that inhibited 50% of cell viability.

### 2.3. Experimental Design and Grouping

According to the IC50 results, two treatment groups were selected for each cell line for the experimental design. GC1-C and GC2-C were cultured under the above conditions without paclitaxel. As the negative control, these were used to establish the baseline ER stress protein expression. Paclitaxel (43 nM for GC1 and 34 nM for GC2) was added to these experimental groups, namely GC1-P and GC2-P, and the cells were incubated for 24 h. Necrosis or cell death was avoided by selecting this time point, because the preliminary assays suggested that within this period there was a significant cellular response. After treatment, all cells were collected and processed for immunocytochemical analysis to determine the expression of the ER stress marker.

### 2.4. Immunocytochemical Procedure

Following treatment, the cells were carefully washed with phosphate-buffered saline (PBS) and then fixed in 4% paraformaldehyde, for 15 min at room temperature, to preserve the cells’ structural form. The cells were then permeabilized by treatment with 0.1% Triton X-100 in PBS for 10 min to permit access of the antibody to the intracellular epitopes. The cells were incubated with 5% bovine serum albumin (BSA) in PBS for 1 h to block non-specific antibody binding [19]. The following primary antibodies were used in this study: anti-GRP78 (Rabbit polyclonal, Bioss, Cat# bs-1211R, 1:200 dilution), anti-p-PERK (Rabbit polyclonal, Bioss, Cat# bs-3330R, 1:200 dilution), and anti-p-eIF2α (Rabbit polyclonal, Bioss, Cat# bs-1328R, 1:200 dilution) and the cells were incubated overnight at 4 °C. To assess non-specific binding, negative control slides were prepared by omitting the primary antibody step while keeping all other incubation and detection conditions identical. These controls exhibited no DAB signal, confirming the specificity of the primary antibody labeling. Nuclear visualization was performed with Mayer’s hematoxylin staining. The samples kept in the dark were then covered with coverslips, mounted using antifade mounting medium, and stored until imaging. Images were taken using an Axio observer microscope (Zeiss, Jena, Germany) under standardized exposure conditions to retain the consistency between samples. Following immunostaining, semi-quantitative image analysis was performed using ImageJ software (version Fiji) (National Institutes of Health, USA). Five randomly selected fields per sample were captured under identical exposure settings using the Axio observer microscope (Zeiss, Jena, Germany). The mean intensity of immunoreactivity for each marker (GRP78, p-PERK, and p-eIF2α) was measured and statistically compared across groups. This approach enabled objective quantification of protein expression and minimized subjective interpretation. Two independent histologists blinded to the treatment groups evaluated the samples to ensure reproducibility and reduce observer bias [20].

### 2.5. Statistical Analysis

All experimental procedures were performed in biological triplicate. Within each biological replicate, technical triplicates (e.g., three wells per condition) were included for quantification and statistical analysis. Quantitative data were expressed as the median ± range. Prior to statistical analysis, data normality was assessed using the Shapiro–Wilk test. As the data did not meet the assumptions of normal distribution, non-parametric Mann–Whitney U tests were applied to compare differences between groups. All statistical analyses were performed using GraphPad Prism (version 10.4.1), and *p*-values < 0.05 were considered statistically significant.

## 3. Results

**Figure 1:**
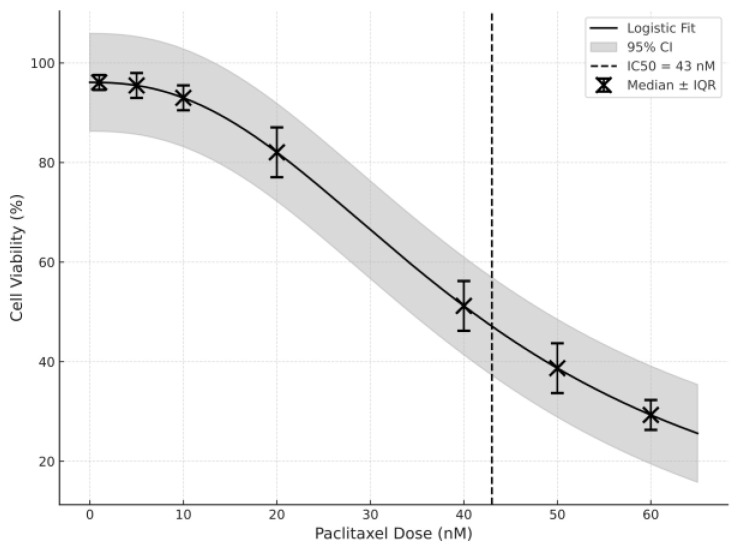
Graph of the IC50 value of paclitaxel used on the GC1 cell line (data shown as median with IQR; non-linear regression with 95% confidence intervals included).

**Figure 2:**
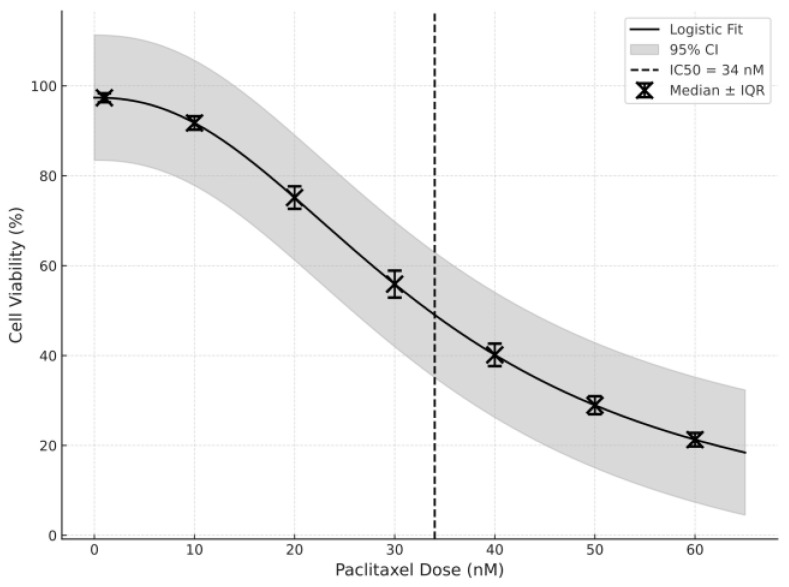
Graph of the IC50 value of paclitaxel used on the GC2 cell line (data shown as median with IQR; non-linear regression with 95% confidence intervals included).

The IC50 values for the cytotoxic potential of paclitaxel were obtained for both GC1 and GC2, which was assessed using MTT assays. As demonstrated in Figure 1, which shows the cell viability curves, the cell viability decreased in a dose-dependent manner at higher concentrations of paclitaxel. Thus, the IC50 value was calculated to be 34 nM for GC2 cells (Figure 1) and 43 nM for GC1 cells (Figure 2).

Therefore, this result suggests that GC2 cells are more responsive to paclitaxel than GC1. This conclusion can also be seen in the steeper slope of the viability curve of GC2 cells, which implies that spermatocytes are more vulnerable to paclitaxel-induced cytotoxicity than spermatogonia. This is a stage-specific response, suggesting either a differential protective capacity or that different stages in the spermatogenic lineage have different capacities to adapt to stress.

There were distinct morphological changes after paclitaxel treatment. Cells in both the GC1-C and GC2-C groups maintained a healthy morphology, well-spread cytoplasm, intact nuclei, and normal intercellular spacing. The cells were round or oval in shape with smooth edges and roundly covered the surface of the culture dish, while the GC2 cells had a slightly larger cytoplasm with a typical pre-meiotic feature.

However, both the paclitaxel-treated groups, GC1-P and GC2-P, were significantly morphologically degenerated. The GC1-P cells appeared visibly shrunken, with irregular cellular boundaries that reduced the cytoplasmic volume. Some nuclear fragmentation and condensation (signals of early apoptosis) were present too. The morphological damage in the GC2-P cells was even more pronounced. The cells looked smaller, detached more readily from the surface, and contained very condensed chromatin. The differences between the GC1-P and GC2-P groups imply that the GC1-P spermatocytes are more structurally and functionally disrupted than the GC2-P spermatogonia.

**Figure 3:**
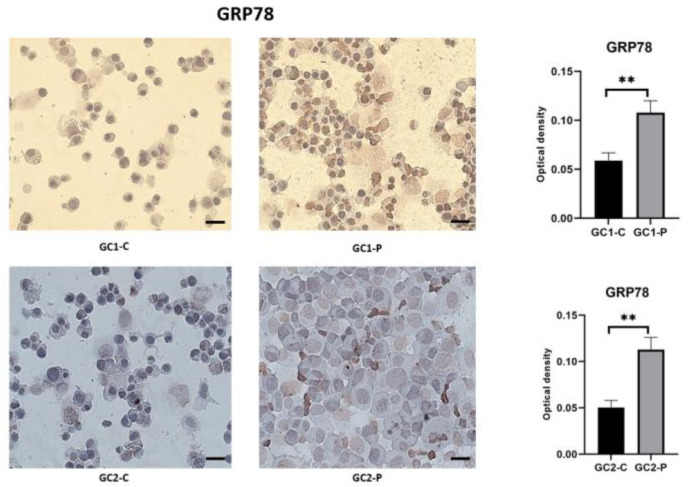
GRP78 immunohistochemical staining images and statistical results (** *p* < 0.05; data shown as median with IQR; statistical analysis conducted using the Mann–Whitney U test via GraphPad Software). All images were captured using a Zeiss Axio Observer microscope (Zeiss, Jena, Germany) at 40× magnification with standardized exposure settings. Optical density values were calculated using ImageJ software. Quantification was based on optical density measurements from five randomly selected fields per sample. Data represent three independent biological replicates. Scale bar: 50 μm.

Paclitaxel treatment strongly induced the master chaperone protein GRP78 (relevant in the detection of unfolded proteins in the ER), which is an indicator of ER stress. The relative GRP78 expression across all groups is shown in Figure 3, using immunocytochemistry images. To set a baseline for homeostasis, in the control cells (GC1-C and GC2-C), the GRP78 staining was weak and diffusely distributed within the cytoplasm.

The induction of GRP78 immunoreactivity was high in the treated groups. The staining became more localized within the perinuclear ER regions and denser in GC1-P cells, suggesting more active chaperone activity. Further strengthening this notion, a significant number of GC2-P cells displayed very strong GRP78 staining, with its distribution spread across the cytoplasm, and there was brighter intensity than in the GC2 cells. Our findings indicate that paclitaxel induces significant ER stress when the protein-folding capacity of the ER is exceeded, which was more pronounced than in GC2 cells (Figure 3).

**Figure 4:**
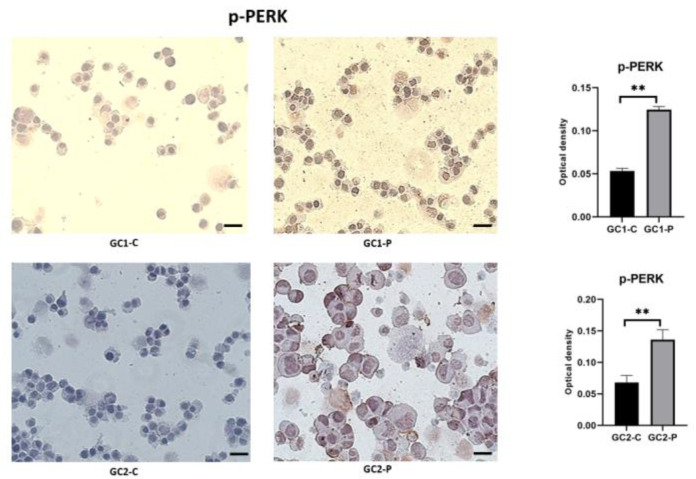
p-PERK immunohistochemical staining images and statistical results (** *p* < 0.05; data shown as median with IQR; statistical analysis conducted using the Mann–Whitney U test via GraphPad Software). All images were captured using a Zeiss Axio Observer microscope at 40× magnification with standardized exposure settings. Optical density values were calculated using ImageJ software. Quantification was based on optical density measurements from five randomly selected fields per sample. Data represent three independent biological replicates. Scale bar: 50 μm.

When misfolded proteins are accumulated, one of the two ultimate sensors of ER membrane stress, PERK, becomes activated. The stains of the p-PERK across the experimental conditions are depicted in Figure 4. p-PERK expression was minimal in the control GC1 and GC2 cells and displayed only weak localization around the ER.

After paclitaxel treatment, the expression of p-PERK was also strongly elevated in both cell lines. The nuclear PERK immunoreactivity became prominent in GC1-P cells, a mark of PERK activation and transduction of ER stress signaling. The p-PERK levels in GC2-P were even higher, and the punctate staining was dense and spread throughout the cytoplasm. This pattern demonstrates PERK oligomerization and activation, key for global protein translation to stop and for adaptive responses to begin. Finally, the elevated activation of p-PERK in the GC2-P cells is in line with their increased sensitivity to ER stress (Figure 4).

**Figure 5:**
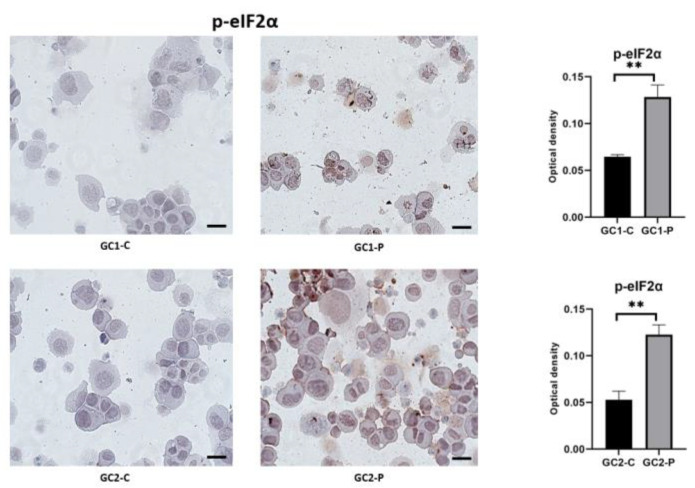
p-eIF2α immunohistochemical staining images and statistical results (** *p* < 0.05; data shown as median with IQR; statistical analysis conducted using the Mann–Whitney U test via GraphPad Software). All images were captured using a Zeiss Axio Observer microscope at 40× magnification with standardized exposure settings. Optical density values were calculated using ImageJ software. Quantification was based on optical density measurements from five randomly selected fields per sample. Data represent three independent biological replicates. Scale bar: 50 μm.

Activation of the PERK pathway results in translational suppression, as shown by phosphorylated eIF2α (p-eIF2α). The expression profiles of p-eIF2α are shown in Figure 5. Staining of the control cells was negligible and was in agreement with the unstressed ER environment.

In the GC1-P cells, the p-eIF2α levels significantly increased, with a larger extension of intensity from the perinuclear ER to the cytoplasm. The GC2-P cells expressed the highest levels, again with very widespread and intensely stained translational arrest indicated (Figure 5). This is a protective mechanism in reducing the burden of protein on the cells’ ER but can also result in apoptosis when it continues. The equivalently observed increase in PERK and p-eIF2α in the same groups supports the functional activation of the UPR pathway.

To summarize the results, it was found that the GRP78, PERK, and eIF2α immunoreactivities increased in the GC1-P and GC2-P groups, as compared to the GC1-C and GC2-C groups.

## 4. Discussion

The effect of paclitaxel, a widely used chemotherapeutic agent, on the induction of endoplasmic reticulum (ER) stress in mouse spermatogonium (GC1) and spermatocyte (GC2) cell lines was studied. Strong evidence was presented that paclitaxel exposure results in substantive ER stress in both GC1 and GC2 cells; however, there is a dissimilar response between these two stages of spermatogenesis. More specifically, the immunocytochemical analysis of major ER stress markers, including GRP78, PERK, and phosphorylated eIF2α, showed that paclitaxel treatment activates a robust UPR in the spermatocytes (GC2), which is more pronounced than in spermatogonia (GC1). Our findings support the hypothesis that paclitaxel exerts a cytotoxic effect in spermatogenic cells by triggering ER stress pathways, which connects to the reproductive toxicity reported in spermatogenic cells of male cancer patients undergoing chemotherapy [21,22].

In this study, the IC50 values were measured for the GC1 and GC2 cell lines using an MTT assay to evaluate the cytotoxic effect of paclitaxel. GC2 cells (spermatocytes) were more sensitive to paclitaxel than GC1 cells, and the IC50 values were 34 nM and 43 nM, respectively. The differential sensitivity to paclitaxel in spermatocytes compared with spermatogonia could be due to the fact that spermatocytes have higher mitotic activity, and the metabolic demands of the meiotic stage are greater. These results are consistent with previous studies that have shown the stage-specific sensitivity of germ cells to chemotherapy agents. For example, studies showed that spermatocytes are more sensitive to cytotoxic drugs, possibly because they are in active cell cycle progression, and they require high levels of protein synthesis for meiosis [23,24]. However, spermatogonia that are in the mitotic phase may have a greater capacity to repair DNA damage or activate survival pathways, or they may be less sensitive to paclitaxel [25].

After paclitaxel treatment, there were clear morphological changes, due to cytotoxicity, in both GC1 and GC2 cells. Healthy morphology was maintained in the control cells, with the well-spread cytoplasm of GC1 and GC2, with intact nuclei, whereas the paclitaxel-treated cells (GC1-P and GC2-P) were dramatically degenerated, with shrinkage, irregular boundaries, and chromatin condensation. The changes observed are consistent with the initiation of apoptosis, typical in cells stressed by chemotherapy [26]. The GC1 cells (spermatogonia) with the radiolabeled PCBs showed more pronounced morphological damage than the GC2 cells (spermatocytes). The IC50 data were also consistent with this observation, suggesting that spermatocytes are more susceptible to paclitaxel-induced cytotoxicity. Previous studies have shown that chemotherapeutic agents such as paclitaxel cause apoptosis and autophagy in spermatogenic cells, resulting in testicular atrophy and male infertility in patients [27,28]. The higher metabolic activity and complexity of meiotic progression in spermatocytes increases their vulnerability to DNA damage and ER stress, resulting in the increased sensitivity of spermatocytes to paclitaxel-induced apoptosis.

The central purpose of this study was to identify whether paclitaxel exposure induced ER stress pathways. The immunocytochemical analysis showed a significant increase in the expression of GRP78, PERK, and phosphorylated eIF2α, key ER stress markers, in paclitaxel-treated cells in comparison to the controls. GRP78 was significantly upregulated in both GC1 and GC2 cells with more intense staining in the GC2 cells, indicating a higher degree of ER stress in spermatocytes. Under normal conditions, GRP78 acts as a key sensor of ER stress and binds to and inactivates the three primary UPR sensors: PERK, IRE1, and ATF6 [29]. This leads to the accumulation of unfolded or misfolded proteins within the lumen of the ER, which promotes the dissociation of GRP78 from these sensors to allow their activation [30]. The data presented in this study further strengthen the hypothesis that paclitaxel treatment induces significant ER stress by overloading the protein-folding capacity of the ER, as suggested by the upregulation of GRP78 in the paclitaxel-treated cells. With the increased expression of GRP78 in GC2 cells, the spermatocytes are more stressed than the spermatogonia, probably because during meiosis the spermatocytes need to synthesize a higher number of proteins.

We also determined that both the GC1 and GC2 cell lines had their PERK, an ER stress sensor, upregulated after paclitaxel treatment. PERK activation phosphorylates eIF2α, which is a critical step for UPR attenuation of global protein translation so as to decrease the load of the newly synthesized proteins in the ER [31]. The UPR, as a protective mechanism against ER stress, was demonstrated by the fact that the paclitaxel-treated cells showed increased expression of PERK and p-eIF2α, especially in GC2 cells. Activation of the UPR was further supported by the phosphorylation of eIF2α, which was markedly increased in both GC1-P and GC2-P cells. This response seeks to decrease protein synthesis in order to allow the ER to recover from stress by reducing the entry of newly synthesized proteins [32]. Thus, for the GC2 cells, the intense staining of p-eIF2α shows a stronger translational attenuation response, which may indicate that the spermatocytes are experiencing a higher ER overload. Similar to previous reports, this finding is consistent with the knowledge that ER-stress-induced translational inhibition is defined by eIF2α phosphorylation [33].

The results of this study are highly relevant to male reproductive health, most specifically to chemotherapy-induced infertility. Because paclitaxel causes substantial ER stress in spermatogenic cells, it may be a contributing factor to the reproductive toxicity experienced by male cancer patients. Since spermatocytes are more metabolically active, and the demands for protein synthesis are higher, they appear to be more vulnerable to paclitaxel-induced damage. Activation of the UPR in these cells may explain, therefore, how chemotherapy agents disrupt spermatogenesis and cause oligospermia, reduced sperm motility, and infertility [34]. Additionally, this study shows the need to develop fertility-preserving strategies for male cancer patients. Recent developments in molecular biology have raised the possibility of blocking the UPR pathway in order to protect reproductive cells from chemotherapy [35]. For example, pharmacological agents that regulate the ER stress pathways or improve the capacity for protein folding in the ER may enable the protection of spermatogenic cells from chemotoxicity [36].

Another important limitation of our study is the absence of downstream proapoptotic markers (such as CHOP or Caspase-12) or direct apoptosis assays (e.g., Annexin V-FITC/PI, TUNEL, or cleaved Caspase-3). This was a deliberate methodological choice to restrict our analysis to early-stage ER stress signaling events. The goal was to identify whether paclitaxel acts as an upstream ER stress inducer in spermatogenic cells, using well-established early ER markers like GRP78, p-PERK, and p-eIF2α. These markers are known to precede proapoptotic signaling within the unfolded protein response. Although we observed morphological features suggestive of apoptosis (e.g., nuclear condensation), definitive mechanistic conclusions cannot be drawn without further molecular validation. Future studies are warranted to incorporate downstream effectors and functional assays to determine whether ER stress transitions into apoptosis in this cellular context [37,38,39].

A further constraint lies in the exclusive use of immunocytochemistry for evaluating ER stress markers. While this technique provides valuable spatial and morphological context regarding protein expression and localization, it is inherently semi-quantitative. The absence of complementary quantitative analyses such as Western blotting or qRT-PCR limits the precision with which we can interpret the extent of molecular changes induced by paclitaxel. Future investigations should incorporate these molecular techniques to validate and expand upon the current findings. Such approaches would not only corroborate the immunocytochemical results but also offer deeper insight into the transcriptional and translational dynamics of ER stress signaling in spermatogenic cells.

Additionally, a limitation of this study is the evaluation of ER stress responses at a single post-treatment time point (24 h). This time frame was selected based on prior findings indicating that key markers such as GRP78 and p-PERK peak within 12–24 h following paclitaxel exposure. However, ER stress is a dynamic process, and certain early (e.g., GRP78) or late (e.g., CHOP) responses may fall outside this window [40,41]. Including multiple time points in future studies (e.g., 6 h, 12 h, 48 h) could provide a more comprehensive understanding of the temporal profile of paclitaxel-induced ER stress.

## 5. Conclusions

This study is, to our knowledge, the first to investigate paclitaxel-induced ER stress responses in GC1 and GC2 spermatogenic cell lines. While our findings provide important preliminary insights, validation in additional germ cell lines—including human-derived models—is needed to confirm the generalizability and translational relevance of these results.

The objective of this study was to probe the influence of paclitaxel on ER stress in spermatogenic cells utilizing markers of ER stress, including GRP78, PERK, and p-eIF2α. The results showed that paclitaxel treatment induced significant ER stress in both the GC1 and GC2 cell lines. Comparison of the controls versus the cells treated with paclitaxel showed that the cells exposed to paclitaxel had higher levels of ER stress markers (GRP78, PERK, and p-eif2a), indicating that the UPR was triggered in the paclitaxel-treated spermatogenic cells. Specifically, the paclitaxel response of spermatocytes (GC2) was more severe within spermatogenic cells at different stages of development. Furthermore, exposure to paclitaxel induced a large number of morphological changes consistent with apoptotic cell death, including nuclear fragmentation and cytoplasmic condensation. Although our morphological analysis showed features consistent with early apoptosis, such as nuclear condensation, it is important to note that no definitive apoptosis assays were performed. Therefore, the conclusions of this study were framed to reflect the preliminary nature of the data and acknowledge the lack of direct apoptosis measurements. The data generated in this study detail an important set of molecular mechanisms through which paclitaxel is capable of inducing reproductive toxicity. The repeated observation was that paclitaxel’s cytotoxicity in spermatogenic cells is due to its activation of the ER stress response, in particular, the UPR pathway. Our results provide a direction for future studies to establish pharmacologic protection of spermatogenesis during chemotherapy. Further molecular studies of the mechanisms of ER stress and the UPR in spermatogenic cells may lead to more fertility-preserving interventions for male cancer patients undergoing chemotherapy to improve their post-treatment quality of life.

## Data Availability

All data generated or analyzed during this study are included within this article.

## Abbreviations

PERK	Protein Kinase R-like ER Kinase
GC1	Mouse Spermatogonial Cell
CHOP	C/EBP Homologous Protein
ER	Endoplasmic Reticulum
UPR	Unfolded Protein Response
IRE1-	Inositol-requiring Enzyme 1 Alpha
ATF6	Activating Transcription Factor 6
GRP78	Glucose-regulated Protein 78
DMEM	Dulbecco’s Modified Eagle Medium
FBS	Fetal Bovine Serum
MTT	3-(4,5-Dimethylthiazol-2-yl)-2,5-Diphenyltetrazolium Bromide
PBS	Phosphate-buffered Saline
DMSO	Dimethyl Sulfoxide
DAB	3,3′ Diaminobenzidine
SD	Standard Deviation
